# Pacing Strategies in the ‘Athens Classic Marathon’: Physiological and Psychological Aspects

**DOI:** 10.3389/fphys.2018.01539

**Published:** 2018-11-02

**Authors:** Pantelis T. Nikolaidis, Beat Knechtle

**Affiliations:** ^1^Exercise Physiology Laboratory, Nikaia, Greece; ^2^Laboratory of Exercise Testing, Hellenic Air Force Academy, Dekelia, Greece; ^3^Institute of Primary Care, University of Zürich, Zürich, Switzerland; ^4^Medbase St. Gallen Am Vadianplatz, St. Gallen, Switzerland

**Keywords:** endurance exercise, master athletes, maximal oxygen uptake, motivation, performance level, speed

## Abstract

Despite the increased scientific interest in the relationship between pacing and performance in marathon running, little information is available about the association of pacing with physiological and psychological parameters. Therefore, the aim of the present study was to examine the role physical fitness and training characteristics on pacing in the ‘Athens Classic Marathon.’ Finishers in this race in 2017 (women, *n* = 26, age 40.8 ± 9.4 years; men, *n* = 130, age 44.1 ± 8.6 years) were analyzed for their pacing during the race, completed the Motivation of Marathon Scale (MOMS) and performed a series of physiological tests. Women and faster recreational runners adopted a more even pacing. A more even pacing was related with a higher aerobic capacity and lower muscle strength in men, but not in women. Men with more even pacing scored higher in psychological coping, self-esteem, life meaning, recognition and competition than their counterparts with less even pacing. Considering the increasing number of participants in marathon races, these findings might help a wide range of professionals (fitness trainers, physiologists, and psychologists) working with runners to optimize the pacing of their athletes.

## Introduction

An increased scientific interest on pacing in marathon running has been observed during the last 10 years, probably due to the relationship of pacing with performance (Ely et al., 2008; Nikolaidis and Knechtle, 2018). A better pacing strategy could provide elite marathon runners with an economical pathway to significant performance improvements at world-record level (Angus, 2014). Pacing has evolved during the last 50 years, record holders between 1967 and 1988 started significantly faster, although after 25 km, their speed dropped dramatically and was significantly slower than in record holders between 1988 and 2018 (Diaz et al., 2018).

It has been observed that winners in marathons run an even pace throughout the race (Haney and Mercer, 2011; Breen et al., 2018; Diaz et al., 2018), whereas runners of lesser ability slowed as the race progressed, particularly after 20–25 km (Ely et al., 2008). In addition, faster marathoners tend to run at a more consistent pace compared with slower runners (March et al., 2011), whereas slower marathon finishers had greater variability of pace compared to faster marathoner finishers (Haney and Mercer, 2011). It is also known that older and women are better pacers than younger and men, respectively (March et al., 2011). In women, the fastest runners achieved better finishing times relative to their personal best record than their slower peers, who selected unsustainable initial speeds resulting in subsequent significant losses of speed (Renfree and St Clair Gibson, 2013). Recently, the role of sex, age and performance on pacing was confirmed in the ‘New York City Marathon’ (Nikolaidis and Knechtle, 2017, 2018; Breen et al., 2018). For instance, it was observed that women exhibited less variable pacing than men (Breen et al., 2018).

Although the abovementioned studies improved our understanding of the effect of sex, age and performance on pacing, little information is available about the role of physiological and psychological characteristics. On the one hand, it is reasonable to assume that since fast marathon runners had high maximal oxygen uptake, anaerobic threshold (Esteve-Lanao et al., 2017; Takayama et al., 2017), and low body mass, body mass index (BMI), body fat percentage (BF) and leg volume (Tanda and Knechtle, 2013; Salinero et al., 2017), these physiological characteristics would be related to pacing. On the other hand, psychological characteristics might influence decision making related to pacing, and poor decisions would result in final performances inferior to those expected based on personal best times (Renfree and St Clair Gibson, 2013). Furthermore, psychological characteristics, such as motivations, have been shown to correlate with marathon race time (Masters et al., 1993); thus, the estimation of the relationship between pacing and motivations would be of practical relevance for sport psychologists working with marathon runners. Also, pacing might be influenced by an interaction between feedback and previous experience (Micklewright et al., 2010). A better understanding of the physiological and psychological correlates of pacing remains an important issue with practical implications for coaches and fitness trainers working with recreational marathon runners. Therefore, the aim of the present study was to examine the relationship of pacing with physiological and psychological characteristics in recreational marathon runners.

## Materials and Methods

### Study Design and Participants

**FIGURE 1:**
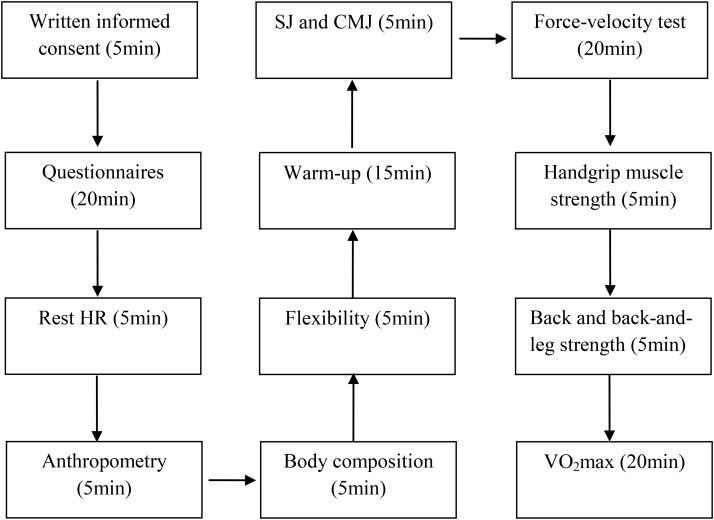
Timeline of the testing session. HR, heart rate; SJ, squat jump; CMJ, countermovement jump; VO_2_max = maximal oxygen uptake.

To examine the relationship of pacing with physiological and psychological aspects, a cross-sectional study design was adopted, where finishers in the ‘Athens Authentic Marathon’ were invited to participate through relevant advertisements in social media specialized in endurance runners. The inclusion criteria were that participants (a) had full pacing data available for the ‘Athens Authentic Marathon’ 2017, (b) were injury-free, (c) did not receive any medication during the experimental session, and (d) had no other reason preventing them from performing the experimental session. After being informed for the benefits and risks of this study, participants completed and signed a written informed consent. The study design was in accordance with the Declaration of Helsinki and was approved by the local institutional review board (Exercise Physiology Laboratory, Nikaia, Greece; Dr. Céline Dewas, head of the board). All experimental procedures were conducted on autumn 2017, a month before the race was performed. Participants were 156 marathon runners fulfilling the abovementioned inclusion criteria (women, *n* = 26; men, *n* = 130). The timeline of the single ∼2 h testing session was presented in Figure 1. Briefly, upon their arrival on the laboratory participants completed questionnaires, were examined for rest heart rate (HR), anthropometry, body composition, flexibility, jumping ability, force-velocity characteristics, isometric muscle strength and aerobic capacity. The rationale for the tests’ selection was to assess those physical fitness components related with both sport performance (e.g., anthropometry and aerobic capacity) and health (e.g., flexibility and muscle strength).

#### The Race

‘Athens Authentic Marathon’ has been hold annually since 1972^1^ in the same route where the first organized marathon was run in 1896 during the first modern Olympic Games (Feldman, 2018). The race was developed with reference to the legend of Pheidippides, who ran 42 km from Marathon to Athens carrying news of victory in battle (Greeks versus Persians) (Leischik, 2015). A unique characteristic of the present race is the large change of elevation (successive increase and decrease of elevation). The race starts at elevation 42 m and finishes at 78 m, with a minimum elevation 7 m at 5–10 km split and a maximum elevation 244 m at 30–35 km split.

#### Pace Range and Coefficient of Variation

Data on split times (5 km, 10 km, …, 40 km and finish) for each participant were downloaded from the official website of the race^2^. We calculated absolute speed (km/h) for each split (0–5 km, 5–10 km, …, 35–40 km, and 40–42 m) using the formula ‘5 km/split time’ for all splits, except the last one where the ‘2.195/split time’ was applied. The coefficient of variation (CV) of splits’ speed was considered as a metric of pacing with lower values indicating a more even pacing and *vise versa*. The average race speed was calculated as ‘42.195/race time.’ In addition to absolute speed, we estimated relative speed (%) for each split using the formula ‘100 × (speed of split – average race speed)/average race speed.’ The relative speeds were used to estimate the split with the minimal and maximal relative speed (%) and we calculated pace range as the difference between maximal and minimal relative speed. In summary, CV and pace range were both used to describe pacing. With regards to the consumption of food and beverages in day of the race, the majority reported that they adopted specific nutrition for the race (68%), whereas 21% that it followed a usual nutrition of a training day, 2% of a resting day or it varied depending on the mood (9%).

### Procedures and Equipment

#### Anthropometry

Height, body mass, and skinfolds were measured with participants in minimal clothing and barefoot. An electronic weighing scale (HD-351; Tanita, Arlington Heights, IL, United States) was employed for measurement of body mass (to the nearest 0.1 kg), a portable stadiometer (SECA Leicester, United Kingdom) for height (0.001 m), and a caliper (Harpenden, West Sussex, United Kingdom) for skinfolds (0.2 mm). BMI was calculated as the quotient of body mass (kg) to height squared (m^2^), and BF was estimated from skinfolds (Parizkova, 1978). Parizkova’s prediction equation of BF has been one of the five equations described in the ‘Kinanthropometry and exercise physiology laboratory manual’ (Eston and Reilly, 2001, p. 27–33) and was selected for the present study as it had the largest number of skinfolds’ sites allowing for a more detailed study of fat distribution through the human body. Fat-free mass (FFM) in kg was calculated as ‘body mass – (body weight ^∗^ BF/100).’

#### Neuromuscular Fitness

The sit-and-reach test (SAR) was used to assess low back and hamstring flexibility (Mayorga-Vega et al., 2014). It was performed on a box providing 15 cm advantage, i.e., the participants scores 15 cm when their reach their toes. Two trials were performed with 1min break and the best was recorded in the nearest 0.5 cm. Intra-class correlation coefficient (ICC) of single measures was 0.981 (95% confidence intervals, CI, 0.975; 0.986). To evaluate isometric muscle strength, the sum of four tests (right and left handgrip test, back test, back-and-leg test) either in absolute or relative to body mass values was used. Although isometric dynamometry was not relevant to sports performance, it was a common measure of muscle strength as component of health-related physical fitness. The handgrip test was performed with participants standing and having their elbow bent at approximately 90°. They were instructed to squeeze the handle of the handgrip dynamometer (Takei, Tokyo, Japan) as hard as possible for 5 s. Two trials were performed for each hand, with a 1-min rest between the trials. The best trial was recorded for each hand (Heyward, 2010). ICC was 0.945 (95% CI, 0.926; 0.959) in both hands. A back strength dynamometer (Takei, Tokyo, Japan) was used for both back strength test and back-and-leg test. The back strength test was performed with participants having their legs and backs straightened to allow the bar to be at the level of the patella, while in the combined back-and-leg test, the chain length on the dynamometer was adjusted so that the participants squatted over the dynamometer with their knees flexed at approximately 30° (Heyward, 2010). All measurements were recorded to the nearest 0.1 kg. The participants performed two trials for each jumping test (squat jump, SJ, and countermovement jump, CMJ) and the best result was recorded (Aragon-Vargas, 2000). There was 1min break between trials and tests. Height of each jump was estimated using the Opto-jump (Microgate Engineering, Bolzano, Italy) and was expressed in the nearest 0.1 cm. ICC was 0.914 (95% CI, 0.885; 0.936) in SJ and 0.951 (95% CI, 0.934; 0.963) in CMJ.

#### Force-Velocity Characteristics

The *F*-*v* test was used to assess Pmax, expressed as W and as W⋅kg^−1^ (rPmax), theoretical maximal velocity (*v*_0_) in revolutions per minute (rpm) and force (*F*_0_) in *N*, and *v*_0_/*F*_0_ was calculated in rpm.N^−1^. This test was performed instead of a running anaerobic test as only laboratory tests were included in the present fitness battery. The participants performed four sprints, each one lasting 7 s, against braking force (2, 3, 4, and 5 kg on a counterbalanced order) on a leg cycle ergometer (Ergomedics 874E, Monark, Sweden), interspersed by 5-min recovery periods. The seat height of the ergometer was adjusted to allow for a slight bend in the knee (approximately 175°) and in accordance with the participant’s satisfaction. Each sprint began with a flying start, i.e., as soon as velocity reached 50 rpm (revolutions per minute), the weight basket was released and the braking force was applied. For each participant an individual linear regression was determined between peak velocity and braking force for each of the four sprints. *F*_0_ and *v*_0_ corresponded to the intercepts with *F* and *v* axes in the *F-v* graph. Pmax was calculated as Pmax = 0.25⋅*F*_0_⋅*v*_0_ (Vandewalle et al., 1985).

#### Aerobic Capacity

The protocol of the graded exercise test to assess aerobic capacity has been recently described in details (Nikolaidis et al., 2018a). Briefly, it consisted of running on a calibrated treadmill with initial speed of 8 km.h^−1^ which was increasing every minute by 1 km.h^−1^ till exhaustion. HR was recorded continuously during the test by Team2 Pro (Polar Electro Oy, Kempele, Finland). Minute ventilation and VO_2_ were recorded by a gas analyzer (Fitmate Pro, Cosmed, Rome, Italy). Plateau of VO_2_ (primary criterion), blood lactate, age-predicted HR_max_ [calculated using Tanaka’s formula, i.e., 208-0.7 × age (Tanaka et al., 2001)] and rate of perceived exertion (RPE) Borg category-ratio scale (Borg, 1998) (secondary criteria) were used as criteria of VO_2_max. Due to the difficulty identifying a plateau of VO_2_, VO_2_ was considered maximal once the secondary criteria were fulfilled. Blood samples were taken 5 min after termination of test [i.e., within the recommended range of 1–8 min (Howley et al., 1995)], and lactate concentration was analyzed (Accutrend, Roche, Germany).

#### Motivation

Participants completed the 56-item Motivations of Marathoners Scales (MOMS) which has been shown internal consistent (Cronbach’s alpha range 0.80–0.93), test–retest reliable (intraclass correlation coefficient range 0.71–0.90), and factorial valid for its subscales (Masters et al., 1993). Each item is scored using a 7-point Likert scale, where participants denote the degree of their agreement with each item-‘reason to run’ ranging from 1 (it is not a reason to run) to 7 (it is a very important reason to run). MOMS has been used in many studies (Zach et al., 2017). It identifies four broad categories or reasons for running (and nine specific themes within these categories); psychological (providing a sense of life meaning, enhancing self-esteem, and psychological coping), achievement (achieving personal goals and competing with other runners), social (desire to receive recognition and approval from others, and the desire to affiliate with other runners), and physical motives (general health orientation and concern about weight) (Hammer and Podlog, 2016).

### Statistical and Data Analysis

Statistical analyses were performed using IBM SPSS v.20.0 (SPSS, Chicago, IL, United States) and GraphPad Prism v. 7.0 (GraphPad Software, San Diego, CA, United States). Normality was examined using Kolmogorov–Smirnov test and visual inspection of normal Q–Q plots. Data were expressed as mean and standard deviation (SD). A between-within subjects analysis of variance (ANOVA) examined the main effect of split and the sex × split interaction on race speed expressed either as km/h or as percentage of the average race speed. The magnitude of differences when using ANOVA was estimated using eta square classified as small (0.010 < η^2^≤ 0.059), medium (0.059 < η^2^≤ 0.138), and large (η^2^ > 0.138) was used (Cohen, 1988). An independent *t*-test examined differences between women and men, and within age groups and performance groups in women. In men, a one-way ANOVA was used for the comparison among age groups and performance groups. A stepwise regression analysis was used to develop prediction equations of pacing for each sex using all independent variables considering the assumptions of Tabachnick and Fidell (2013). Significance was set at *p* < 0.05.

## Results

The women participants (*n* = 26) were 40.8 ± 9.4 years old, mean race speed 9.29 ± 1.21 km/h, and had previously completed 3.6 ± 3.9 marathon races (range 1–20), whereas men (*n* = 130) 44.1 ± 8.6 years old, 10.29 ± 1.87 km/h and 5.4 ± 5.9 marathon races (1–35). The overall profile of the Athens Classic Marathon follows a positive pacing with an end spurt. That is, the speed of runners decreases across the race (with the exception of the split 20–25 km, where the speed increased), whereas an increase is observed in the last split. A large main effect of split on race speed (km/h) was observed (*p* < 0.001, η^2^ = 0.551) with the fastest speed in the 20–25 km split (12.53 ± 2.51 km/h) and the slowest in the 15–20 km split (8.49 ± 1.50 km/h), corresponding to the largest descent and ascent of the race, respectively.

**FIGURE 2:**
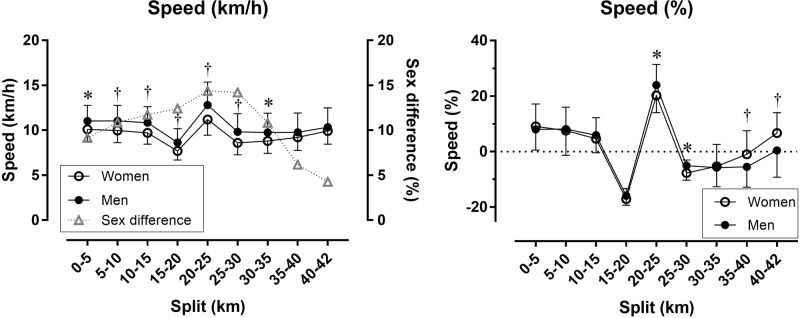
Race speed in absolute **(left)** and relative values **(right)** by sex and split. Error bars represent standard deviations; ^∗^ and ^†^ depict statistical difference between women and men at *p* < 0.05 and *p* < 0.01, respectively. Speed (%) has been calculated as percentage difference of split speed and average race speed.

Women had a smaller pace range than men (*p* = 0.037; 40.6 ± 5.1% versus 43.8 ± 7.5%, respectively), but did not differ in CV (*p* = 0.486; 0.130 ± 0.024 versus 0.134 ± 0.032, respectively). A moderate main effect of sex on race speed (km/h) was found with men faster than women (10.29 ± 1.87 km/h and 9.29 ± 1.21 km/h, respectively). A small sex × split interaction on race speed was shown (*p* = 0.003, η^2^ = 0.031) with the largest sex difference in the 20–25 km (+14.38%) and the smallest in the 40–42 km split (+4.26%) (Figure 2, left). Considering speed (%), a large main effect of split on race speed (%) was found (*p* < 0.001, η^2^ = 0.546) with the fastest speed (%) in the 20–25 km split (+23.35 ± 7.38%) and the slowest in the 15–20 km split (−16.09 ± 3.53%). A small sex × split interaction on race speed (%) (*p* = 0.012, η^2^ = 0.025) with women and men differing at 20–25, 25–30, 35–40, and 40–42 km (Figure 2, right).

In women, no difference was observed in pace range (*p* = 0.088, *d* = 0.69) and CV (*p* = 0.105, *d* = 0.65) between runners younger or older than 41.8 years old (Table 1). In men, no difference was shown in pace range (*p* = 0.653, η^2^ = 0.040) and CV (*p* = 0.899, η^2^ = 0.023) among age groups. In women, no difference was shown between slower and faster runners for pace range (*p* = 0.439) and CV (*p* = 0.080) (Table 2). In men, the pace range did not differ by performance group (*p* = 0.627), but CV did (*p* = 0.033), in which the slowest group had the most variable pacing. The physiological and psychological characteristics of all participants can be seen in Table 3 and their correlations with pacing by sex in Tables 4, 5. In the overall sample, pace range could be predicted by the equation Pace range (%) = 40.157 + 0.462^∗^CMJ-0.806^∗^race speed (*R* = 0.350, SEE = 7.02, *p* < 0.001); in women, Pace range (%) = 79.591–2.362^∗^BMI + 0.652^∗^BF (*R* = 0.756, SEE = 3.48, *p* < 0.001); in men, Pace range (%) = 40.467 + 0.48^∗^CMJ-0.87^∗^race speed (*R* = 0.343, SEE = 7.29, *p* < 0.001). The prediction equations for CV were CV = 0.217–0.009^∗^race speed + 0.002^∗^CMJ-0.001^∗^age (*R* = 0.534, *p* < 0.001, SEE = 0.027) in the total sample; CV = 0.303–0.014^∗^race speed-0.005^∗^BMI + 0.012^∗^goal achievement (*R* = 0.766, *p* < 0.001, SEE = 0.017) in women; CV = 0.285–0.010^∗^race speed–0.001^∗^age (*R* = 0.521, *p* < 0.001, SEE = 0.029) in men.

**Table 1:** Pace range and coefficient of variation (CV) by sex and age group.

	Age (years)	Pace range (%)	CV
**Women (*n* = 26)**			
<41.2 years (*n* = 13)	33.7 ± 6.7	42.3 ± 4.2	0.137 ± 0.024
>41.2 years (*n* = 13)	47.9 ± 5.4	38.9 ± 5.5	0.122 ± 0.022
**Men (*n* = 130)**			
<30 (*n* = 7)	26.7 ± 2.6	45.6 ± 9.3	0.143 ± 0.028
30–35 (*n* = 8)	32.1 ± 1.5	44.8 ± 11.1	0.145 ± 0.053
35–40 (*n* = 25)	37.9 ± 1.6	46.0 ± 9.2	0.138 ± 0.043
40–45 (*n* = 31)	42.4 ± 1.3	44.1 ± 6.2	0.129 ± 0.025
45–50 (*n* = 30)	47.2 ± 1.5	42.9 ± 7.7	0.136 ± 0.035
50–55 (*n* = 17)	52.1 ± 1.3	41.9 ± 5.2	0.131 ± 0.021
55–60 (*n* = 6)	58.2 ± 1.7	43.9 ± 6.6	0.129 ± 0.027
>60 (*n* = 6)	63.6 ± 2.7	40.9 ± 3.9	0.130 ± 0.010

**Table 2:** Pace range and coefficient of variation (CV) by sex and performance group.

	Race time (h:min)	Pace range (%)	CV
**Women (*n* = 26)**			
<4:32 h:min (*n* = 13)	4:42 ± 0:45	39.8 ± 5.8	0.138 ± 0.029
>4:32 h:min (*n* = 13)	4:15 ± 0:29	41.4 ± 4.3	0.121 ± 0.016
**Men (*n* = 127)**			
<3:30 h:min (*n* = 32)	3:10 ± 0:14	43.7 ± 6.8	0.130 ± 0.031
3:30–4:00 h:min (*n* = 31)	3:43 ± 0:08	42.4 ± 4.9	0.126 ± 0.023
4:00–4:30 h:min (*n* = 34)	4:10 ± 0:08	43.8 ± 6.6	0.132 ± 0.028
>4:30 h:min (*n* = 30)	5:06 ± 0:31	44.7 ± 9.3	0.148 ± 0.038

**Table 3:** Descriptive data of physiological and psychological parameters by sex.

	Women (*n* = 26)	Men (*n* = 130)
Age (years)	40.8 ± 9.4	44.1 ± 8.6
Height (cm)	163.4 ± 6.5	176.4 ± 5.8^‡^
Body mass (kg)	58.8 ± 7.5	76.9 ± 9.4^‡^
BMI (kg.m^−2^)	22.0 ± 2.3	24.7 ± 2.6^‡^
BF (%)	20.0 ± 4.8	17.7 ± 4.1^∗^
FFM (kg)	46.9 ± 5.4	63.0 ± 6.1^‡^
VO_2_max (mL.min^−1^.kg^−1^)	37.3 ± 6.3	48.3 ± 8.0^‡^
MAS (km.h^−1^)	13.4 ± 1.3	15.9 ± 1.9^‡^
Lactate (mmol.L^−1^)	8.9 ± 2.6	10.8 ± 3.2^†^
RPE (a.u.)	8.4 ± 0.9	8.8 ± 0.9
Experience (marathon races)	3.6 ± 3.9	5.7 ± 6.4
Pmax (W.kg^−1^)	8.62 ± 1.13	10.36 ± 1.47^‡^
SAR (cm)	24.9 ± 8.4	17.6 ± 8.5^‡^
Isometric strength (kg.kg^−1^ of body weight)	4.06 ± 0.59	5.06 ± 0.78^‡^
SJ (cm)	17.5 ± 3.4	24.3 ± 4.2^‡^
CMJ (cm)	18.1 ± 3.6	25.8 ± 4.8^‡^
Psychological coping	5.4 ± 0.9	4.4 ± 1.4^†^
Self-esteem	5.3 ± 1.1	4.5 ± 1.4^†^
Life meaning	4.3 ± 1.4	3.6 ± 1.5^∗^
Health orientation	5.8 ± 1.0	5.4 ± 1.1^∗^
Weight concern	4.8 ± 1.5	4.1 ± 1.5^∗^
Affiliation	4.1 ± 1.8	3.7 ± 1.5
Recognition	3.0 ± 1.6	2.8 ± 1.5
Competition	3.3 ± 1.7	3.1 ± 1.5
Goal achievement	5.9 ± 0.7	5.0 ± 1.1^†^
Average race speed (km.h^−1^)	9.29 ± 1.21	10.29 ± 1.87^∗^

*^∗^p < 0.05, ^†^p < 0.01, ^‡^p < 0.001. BMI, body mass index; BF, body fat percentage; FFM, fat-free mass; VO_2_max, maximal oxygen uptake; MAS, maximal aerobic speed; RPE, rate of perceived exertion; Pmax, maximal anaerobic power; SAR, sit-and-reach test; SJ, squat jump; CMJ, countermovement jump.*

**Table 4:** Correlations (Pearson *r*) of total pacing range and coefficient of variation of race speed with physiological characteristics.

Variables	Total pacing range		Coefficient of variation	
	Women	Men	Women	Men
Age	−0.21	−0.16	−0.21	−0.09
Height	−0.06	0.03	−0.10	0.04
Body mass	−0.56^†^	0.03	−0.42^∗^	0.21^∗^
BMI	−0.64^‡^	0.02	−0.43^∗^	0.23^†^
BF	−0.16	0.07	−0.04	0.26^†^
FFM	−0.51^†^	<0.01	−0.43^∗^	0.13
VO_2_max	−0.03	−0.05	0.08	−0.20^∗^
MAS	−0.21	<0.01	−0.34	−0.16
Lactate	0.16	0.16	0.31	0.08
RPE	−0.28	<0.01	−0.20	−0.04
Experience	−0.14	−0.07	−0.22	−0.04
Pmax	0.18	0.07	−0.05	−0.03
SAR	−0.28	0.09	−0.19	<0.01
Isometric strength	0.25	−0.05	0.16	−0.17
SJ	−0.10	0.23^†^	−0.07	0.15
CMJ	−0.09	0.28^†^	0.03	0.20^∗^
Average race speed	0.09	−0.17	−0.38	−0.45^‡^

*^∗^p < 0.05, ^†^p < 0.01, ^‡^p < 0.001. A negative correlation indicates that the variable is related with a more even pacing, whereas a positive correlation suggests a more variable pacing. BMI, body mass index; BF, body fat percentage; FFM, fat-free mass; VO_2_max, maximal oxygen uptake; MAS, maximal aerobic speed; RPE, rate of perceived exertion; Pmax, maximal anaerobic power; SAR, sit-and-reach test; SJ, squat jump; CMJ, countermovement jump.*

**Table 5:** Correlations (Pearson *r*) of total pacing range and coefficient of variation of race speed with psychological characteristics.

Variables	Total pacing range		Coefficient of variation	
	Women	Men	Women	Men
Psychological coping	−0.34	−0.07	−0.22	−0.04
Self-esteem	−0.02	0.10	−0.20	−0.07
Life meaning	−0.13	−0.09	−0.22	−0.02
Health orientation	−0.16	−0.06	0.05	−0.02
Weight concern	−0.15	−0.09	−0.06	0.06
Affiliation	−0.14	−0.03	−0.22	0.07
Recognition	−0.08	−0.13	−0.22	<0.01
Competition	0.14	−0.17	<0.01	−0.09
Goal achievement	0.13	−0.14	0.12	−0.14

*A negative correlation indicates that the variable is related with a more even pacing, whereas a positive correlation suggests a more variable pacing.*

## Discussion

The main findings of the present study were that (a) women, older and faster recreational runners adopted a more even pacing, (b) a more even pacing was related with a higher aerobic capacity and lower muscle strength in men, but not in women, and (c) men with more even pacing scored higher in psychological coping, self-esteem, life meaning, recognition and competition than their counterparts with less even pacing. Overall, participants adopted a positive pacing and presented an end spurt, which was in agreement with the pattern of pacing observed in marathon running (Abbiss and Laursen, 2008). An exception in this pattern was found in the 20–25 km, i.e., an increase of speed, which should be attributed to the large change of altitude (ascent). A positive pacing and an end spurt have been already shown in the ‘New York City Marathon’ (Santos-Lozano et al., 2014; Breen et al., 2018; Nikolaidis and Knechtle, 2018).

### Sex

Women had a more even pacing presenting both smaller decrease and variation of speed across race than men. This finding was in agreement with previous studies in the ‘New York City Marathon’ (Nikolaidis and Knechtle, 2017), Olympic and IAAF World Championship marathons (Hanley, 2016) and 14 United States marathons (Deaner et al., 2015a). An explanation of the sex difference in pacing might be sex differences in physiology and decision making (Deaner et al., 2015b; Sandbakk et al., 2018). Moreover, the analysis of the sport-specific motivations showed that women outscored men in six out of nine specific themes (psychological coping, self-esteem, life meaning, health orientation, weight concern and goal achievement) and there was no sex difference in the other three (affiliation, recognition and competition) indicating that women were more motivated. In addition, the sex difference in pacing might be attributed to the more rapid depletion of glycogen in men than in women (Sandbakk et al., 2018). On the other hand, psychological factors might include the higher level of competitiveness (Ogles and Masters, 2003) and intention to take risks in men than in women (Deaner et al., 2015b).

### Age

Although smaller scores of pace range and CV were observed in the older groups the differences among age groups did not reach statistical significance. The disagreement of this finding with previous research should be attributed to differences in sample sizes. It should be noted that previous studies showing that older runners adopted a more even pacing than their younger counterparts were conducted in very large dataset, e.g., *n* = 298,082 (Nikolaidis and Knechtle, 2017). Nevertheless, the more even pacing in older runners, observed in previous research (March et al., 2011), might be due to age-related differences in psychological characteristics. It has been observed that older athletes exhibited more emotional stability than their younger counterparts (Nikolaidis et al., 2018b) and this might assist the former adopting a more even pacing. Moreover, it has been shown that the perception of fatigue presented distinct temporal characteristics across a race, i.e., the runners adjusted their perception depending on the proportion of exercise time that remained (Faulkner et al., 2008). In turn, the perception of exercise-induced fatigue has been observed to increase with age, which might explain the adoption of a more even pacing by older runners (Groslambert and Mahon, 2006).

### Performance

The faster race speed was related to a more even pacing, which was in agreement with previous studies (Ely et al., 2008; Haney and Mercer, 2011; March et al., 2011). For instance, it has been observed that the fastest runners adopted a more even pacing in Japanese Women’s championships (Ely et al., 2008) and in Midwestern United States marathon (March et al., 2011), and had lower variability of pace (Haney and Mercer, 2011; Santos-Lozano et al., 2014). An interpretation of the effect of performance on pacing might be that faster runners adopt more realistic race in contrast to the unsustainable initial speeds of their slower counterparts (Renfree and St Clair Gibson, 2013).

### Physiology

Pacing was related to aerobic capacity and muscle strength in men. However, the small magnitude of their correlations indicated that their relationship was likely mediated by race time. It has been shown that half of the variance in pacing was accounted for by the variance in race time (Haney and Mercer, 2011). In turn, race time could be predicted by aerobic capacity (Till et al., 2016; Salinero et al., 2017). As shown in the present study, aerobic capacity was related inversely with variation in pacing in men, i.e., the higher the aerobic capacity the less variable the pacing. An explanation of this relationship might be that a high aerobic capacity indicated both high VO_2_max, anaerobic threshold and running economy (Gordon et al., 2017). That is, a high aerobic capacity might assist marathon runners maintaining their race speed by preventing fatigue (Sjodin and Svedenhag, 1985; Coyle, 1995).

On the other hand, muscle strength (CMJ) in the present study was related with pace range and variability, i.e., the higher the CMJ, the higher the pace range and more variable the pacing. A more even pacing might be related with less muscle strength, likely due to the relationship of muscle strength with body mass and body composition (Raymond-Pope et al., 2018). For instance, it has been found previously that marathoners had ∼13 and ∼24 cm lower CMJ, and 7 and 14 kg lower body mass than middle distance runners and sprinters, respectively (Vuorimaa et al., 1996). In addition, CMJ of the participants in the present study was in agreement with previous research showing that both competitive and recreational marathon runners had moderate leg muscle strength (Vuorimaa et al., 1996; Petersen et al., 2007). Thus, the observed relationship of pacing with CMJ was not a surprising finding considering the moderate CMJ in marathon runners. Considering flexibility, no relationship was observed with pacing. Although high score in SAR has been shown to relate with low running economy (Jones, 2002; Trehearn and Buresh, 2009), SAR did not correlate with any of pacing indices (pacing range or CV).

With regards to anthropometric characteristics and body composition, the correlates of pacing varied by sex. In men, an increased body mass, BMI and BF was related with a more variable pacing. An interpretation of this finding might be that low values of these anthropometric characteristics were previously observed in the fast marathon runners (Tanda and Knechtle, 2013; Gordon et al., 2017; Salinero et al., 2017), who in turn presented less variable pacing. However, an opposite trend was shown in women, i.e., a more even pacing was related with increased body mass, BMI and FFM. It has been found previously that a fast marathon race time in women was related with low body mass and BMI (Schmid et al., 2012) and it would be expected to observed similar relationship as in men.

### Psychology

Considering the psychological aspects, no correlation was observed between pacing and motivations; however, a high goal achievement in women was predictor of high variation in speed across race. The research hypothesis that since motivations were related with marathon race time (Masters et al., 1993), they would also relate with pacing was not confirmed. An interpretation of the limited role of motivations on pacing might be that other psycho-social factors masked motivations (Renfree et al., 2015; Schiphof-Godart and Hettinga, 2017). Furthermore, psychological factors -other than motivations- specific to the race might influence decision making of runners with regards to the distribution of energy across race (Renfree and St Clair Gibson, 2013).

### Limitations, Strength and Practical Applications

Although the findings of the present study provided novel insight in the physiological and psychological aspects of pacing in marathon race, they should be generalized with caution to races especially with smaller changes of elevation that might impact the physiological and motivational demands. In addition, it was acknowledged that differences among studies on marathon’s pacing might result from the existence of several definitions. We used the pace range (Breen et al., 2018) and CV (Santos-Lozano et al., 2014) as two measures of pacing, whereas other measures included the difference between the 0–5 and 35–40 km split times (Ely et al., 2008), mean speed of the last 9.7 km divided by that of the first 32.5 km (March et al., 2011), mean speed of the last 12.2 km divided by that of the first 30 km (Trubee et al., 2014), change of speed from the first to the second half (Deaner et al., 2015a). On the other hand, its strength was its originality as it was the first to investigate these aspects of pacing in the Athens Classic Marathon. It should be highlighted that most of the above-mentioned studies were conducted on United States marathon races. Furthermore, it was acknowledged that the smaller number of women participants did not allow analyzing the aspects of age and performance using the same methodological approach as in men.

These results would be expected to have practical implications for sports scientists, physiologists, psychologists, and other professionals working with marathon runners in order to increase the odds to optimize pacing. Since marathon race time has been shown to correlate with pacing (i.e., the more even the pacing, the faster the race time) (Nikolaidis and Knechtle, 2017; Breen et al., 2018), recreational marathon runners should focus on physiological and psychological correlates of pacing to achieve less variation in their speed across a race. Training approaches to achieve this goal should assist in the increase of aerobic capacity, whereas nutritional strategies should focus in lowering body mass and BF.

## Conclusion

In summary, the results of this study confirmed the effect of sex, age and performance on pacing, whereas what was novel was the variation of physiological and psychological correlates of pacing by sex and the development of prediction equations of pacing. These findings were of practical relevance for recreational marathon runners and practitioners working with them considering the relationship between pacing and race time. It would be recommended that future research should verify these findings in flat marathon races.

## Author Contributions

PN and BK designed the study and collected the data. PN and BK contributed by writing and editing a part of manuscript. PN and BK contributed by reviewing and editing the manuscript. All authors read and approved the final manuscript.

## Conflict of Interest Statement

The authors declare that the research was conducted in the absence of any commercial or financial relationships that could be construed as a potential conflict of interest.

## Footnotes

1 www.athensauthenticmarathon.gr
