# Corrigendum: Pacing Strategies in the ‘Athens Classic Marathon’: Physiological and Psychological Aspects

**DOI:** 10.3389/fphys.2019.01409

**Published:** 2019-11-15

**Authors:** Pantelis T. Nikolaidis, Beat Knechtle

**Affiliations:** ^1^Exercise Physiology Laboratory, Nikaia, Greece; ^2^Laboratory of Exercise Testing, Hellenic Air Force Academy, Dekelia, Greece; ^3^Institute of Primary Care, University of Zürich, Zürich, Switzerland; ^4^Medbase St. Gallen Am Vadianplatz, St. Gallen, Switzerland

**Keywords:** endurance exercise, master athletes, maximal oxygen uptake, motivation, performance level, speed

In the original article, there was a mistake in the calculation of split times; the fourth split is “15–21.1 km” instead of “15–20 km,” and the fifth split is “21.1–25 km” instead of “20–25 km.” Based on the corrected split times, the following corrections have been made:

## Results

The women participants (*n* = 26) were 40.8 ± 9.4 years old, mean race speed 9.29 ± 1.21 km/h, and had previously completed 3.6 ± 3.9 marathon races (range 1–20), whereas men (*n* = 130) 44.1 ± 8.6 years old, 10.29 ± 1.87 km/h and 5.4 ± 5.9 marathon races (1–35). The overall profile of the Athens Classic Marathon follows a positive pacing with an end spurt. That is, the speed of runners decreases across the race, whereas an increase is observed in the last split. A large main effect of split on race speed (km/h) was observed (*p* < 0.001, η^2^ = 0.376) with the fastest speed in the 0–5 km split (10.87 ± 1.70 km/h) and the slowest in the 30–35 km split (9.59 ± 2.06 km/h).

Women had similar pace range (*p* = 0.399; 24.8 ± 10.8% versus 22.3 ± 14.0%, respectively) and CV (*p* = 0.485; 0.086 ± 0.040 versus 0.079 ± 0.049, respectively) as men. A moderate main effect of sex on race speed (km/h) was found with men faster than women (10.29 ± 1.87 km/h and 9.29 ± 1.21 km/h, respectively). A small sex × split interaction on race speed was shown (*p* = 0.013, η^2^ = 0.025) with the largest sex difference in the 21.1–25 km (+14.37%) and the smallest in the 40–42 km split (+4.26%) (Figure 2, left). Considering speed (%), a large main effect of split on race speed (%) was found (*p* < 0.001, η^2^ = 0.371) with the fastest speed (%) in the 0–5 km split (+8.21 ± 9.02%) and the slowest in the 30–35 km split (−5.72 ± 7.00%). A small sex × split interaction on race speed (%) (*p* = 0.015, η^2^ = 0.024) with women and men differing at 21.1–25, 25–30, 35–40, and 40–42 km (Figure 2, right).

**Figure 2:**
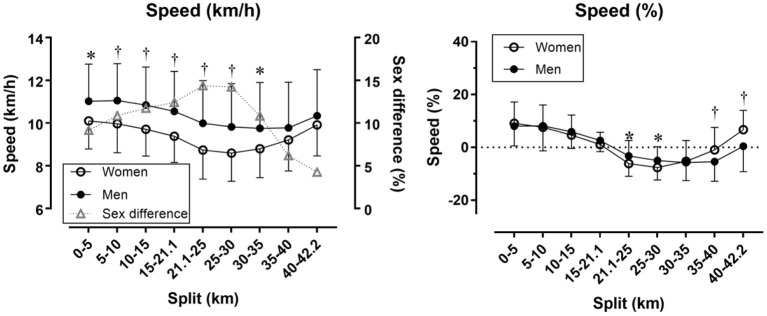
Race speed in absolute **(left)** and relative values **(right)** by sex and split. Error bars represent standard deviations; ^*^ and †depict statistical difference between women and men at *p* < 0.05 and *p* < 0.01, respectively. Speed (%) has been calculated as percentage difference of split speed and average race speed.

In women, no difference was observed in pace range (*p* = 0.185, *d* = 0.54) and CV (*p* = 0.219, *d* = 0.49) between runners younger or older than 41.8 years old (Table 1). In men, no difference was shown in pace range (*p* = 0.721, η^2^ = 0.035) and CV (*p* = 0.619, η^2^ = 0.042) among age groups. In women, the faster runners had smaller pace range (*p* = 0.002) and CV (*p* = 0.003) than their slower counterparts (Table 2). In men, pace range (*p* = 0.003, η^2^ = 0.109) and CV differed by performance group (*p* = 0.006, η^2^ = 0.097), where the slowest group had the most variable pacing. The physiological and psychological characteristics of all participants can be seen in **Table 3** and their correlations with pacing by sex in Tables 4, 5. In the overall sample, pace range could be predicted by the equation Pace range (%) = 56.160–4.44^*^race speed + 0.469^*^CMJ (*R* = 0.550, SEE = 11.4, *p* < 0.001); in women, Pace range (%) = 73.657–7.312^*^race speed + 1.016^*^CMJ (*R* = 0.705, SEE = 8.17, *p* < 0.001); in men, Pace range (%) = 52.889–4.228^*^race speed + 0.503^*^CMJ (*R* = 0.571, SEE = 11.64, *p* < 0.001). The prediction equations for CV were CV = 0.271–0.02^*^race speed −0.001^*^age + 0.001^*^VO_2_max (*R* = 0.608, *p* < 0.001, SEE = 0.039) in the total sample; CV = 0.220–0.026^*^race speed + 0.026^*^isometric strength (*R* = 0.690, *p* < 0.001, SEE = 0.031) in women; CV = 0.316–0.017^*^race speed – 0.001^*^age (*R* = 0.590, *p* < 0.001, SEE = 0.041) in men.

**Table 1:** Pace range and coefficient of variation (CV) by sex and age group.

	**Age (years)**	**Pace range (%)**	**CV**
**Women (*****n*** **=** **26)**			
<41.2 years (*n* = 13)	33.7 ± 6.7	27.6 ± 11.7	0.096 ± 0.045
>41.2 years (*n* = 13)	47.9 ± 5.4	21.9 ± 9.5	0.076 ± 0.034
**Men (*****n*** **=** **130)**			
<30 (*n* = 7)	26.7 ± 2.6	24.9 ± 13.1	0.091 ± 0.046
30–35 (*n* = 8)	32.1 ± 1.5	24.1 ± 18.6	0.092 ± 0.071
35–40 (*n* = 25)	37.9 ± 1.6	22.8 ± 19.1	0.079 ± 0.065
40–45 (*n* = 31)	42.4 ± 1.3	18.3 ± 10.0	0.063 ± 0.037
45–50 (*n* = 30)	47.2 ± 1.5	25.3 ± 15.3	0.089 ± 0.053
50–55 (*n* = 17)	52.1 ± 1.3	22.6 ± 9.2	0.080 ± 0.036
55–60 (*n* = 6)	58.2 ± 1.7	19.6 ± 9.8	0.071 ± 0.037
>60 (*n* = 6)	63.6 ± 2.7	22.7 ± 9.0	0.080 ± 0.037

**Table 2:** Pace range and coefficient of variation (CV) by sex and performance group.

	**Race time (h:min)**	**Pace range (%)**	**CV**
**Women (*****n*** **=** **26)**			
<4:32 h:min (*n* = 13)	4:15 ± 0:29	18.6 ± 7.4	0.064 ± 0.028
>4:32 h:min (*n* = 13)	4:42 ± 0:45	31.0 ± 10.2	0.108 ± 0.039
**Men (*****n*** **=127)**			
<3:30 h:min (*n* = 32)	3:10 ± 0:14	19.1 ± 12.5	0.069 ± 0.047
3:30–4:00 h:min (*n* = 31)	3:43 ± 0:08	18.7 ± 9.8	0.068 ± 0.038
4:00–4:30 h:min (*n* = 34)	4:10 ± 0:08	20.7 ± 12.3	0.072 ± 0.044
>4:30 h:min (*n* = 30)	5:06 ± 0:31	29.9 ± 16.6	0.105 ± 0.057

**Table 4:** Correlations (Pearson *r*) of total pacing range and coefficient of variation of race speed with physiological characteristics.

**Variables**	**Total pacing range**		**Coefficient of variation**	
	**Women**	**Men**	**Women**	**Men**
Age	−0.15	<0.01	−0.14	−0.01
Height	−0.05	0.01	−0.03	0.02
Body mass	−0.13	0.28^†^	−0.17	0.28^†^
BMI	−0.12	0.33^‡^	−0.17	0.32^‡^
BF	0.03	0.32^‡^	−0.04	0.32^‡^
FFM	−0.17	0.18^*^	−0.18	0.18^*^
VO_2_max	<0.01	−0.25^†^	0.10	−0.24^†^
MAS	−0.38	−0.27^†^	−0.29	−0.26^†^
Lactate	0.26	0.04	0.23	0.04
RPE	−0.06	−0.09	<0.01	−0.08
Experience	−0.14	−0.01	−0.13	0.01
Pmax	−0.11	−0.08	−0.11	−0.08
SAR	−0.04	−0.07	−0.07	−0.06
Isometric strength	0.02	−0.21^*^	0.10	−0.20^*^
SJ	−0.04	0.09	−0.01	0.09
CMJ	−0.08	0.09	0.10	0.10
Average race speed	−0.58^†^	−0.55^‡^	−0.51^†^	−0.54^‡^

* *p < 0.05*,

† *p < 0.01*,

‡ *p < 0.001. A negative correlation indicates that the variable is related with a more even pacing, whereas a positive correlation suggests a more variable pacing. BMI, body mass index; BF, body fat percentage; FFM, fat-free mass; VO_2_max, maximal oxygen uptake; MAS, maximal aerobic speed; RPE, rate of perceived exertion; Pmax, maximal anaerobic power; SAR, sit-and-reach test; SJ, squat jump; CMJ, countermovement jump*.

**Table 5:** Correlations (Pearson *r*) of total pacing range and coefficient of variation of race speed with psychological characteristics.

**Variables**	**Total pacing range**		**Coefficient of variation**	
	**Women**	**Men**	**Women**	**Men**
Psychological coping	−0.10	0.02	−0.03	0.02
Self-esteem	−0.27	−0.03	−0.24	−0.02
Life meaning	−0.17	0.04	−0.15	0.06
Health orientation	0.13	0.02	0.11	0.01
Weight concern	−0.07	0.14	−0.04	0.14
Affiliation	−0.20	0.11	−0.26	0.12
Recognition	−0.26	0.07	−0.28	0.09
Competition	−0.13	−0.03	−0.10	−0.02
Goal achievement	0.04	−0.13	0.08	−0.13

*A negative correlation indicates that the variable is related with a more even pacing, whereas a positive correlation suggests a more variable pacing*.

The authors declare for these errors and state that they do not change the scientific conclusions of the article in any way. The original article has been updated.

